# Chronic pain in the aging adult with sickle cell disease

**DOI:** 10.3389/fpain.2026.1907108

**Published:** 2026-09-22

**Authors:** Keesha Powell-Roach, Ugochi O. Ogu

**Affiliations:** 1 Center for Sickle Cell Disease, University of Tennessee Health Science Center, Memphis, TN, United States; 2 Rutgers Biomedical and Health Sciences, Rutgers University, Division of Nursing Science, School of Nursing, Newark, NJ, United States; 3 Department of Health Promotion and Disease Prevention, University of Tennessee Health Science Center, College of Nursing, Memphis, TN, United States; 4 Division of Hematology, University of Tennessee Health Science Center, Memphis, TN, United States

**Keywords:** aging, analgesics, chronic pain, health disparities, neuropathic pain, older adult, pain management, sickle cell disease

## Abstract

Improved survival in sickle cell disease (SCD) has led to a growing population of older adults who experience a high burden of chronic pain. With aging, pain often shifts from episodic vaso-occlusive crises to persistent, multifactorial syndromes driven by cumulative tissue injury, central sensitization, and progressive organ damage. This evolution presents significant clinical challenges, particularly in the context of comorbidities, polypharmacy, and age-related physiological changes. This narrative review examines the epidemiology, pathophysiology, and management of chronic pain in aging adults with SCD. Key contributors to pain include musculoskeletal degeneration, neuropathic mechanisms, and chronic inflammation, compounded by renal, hepatic, and cardiovascular dysfunction. Pharmacological management requires careful consideration of analgesic safety, particularly with opioids, nonsteroidal anti-inflammatory drugs, and adjuvant agents. Nonpharmacological approaches, including rehabilitative and behavioral strategies, are essential components of care. Psychosocial factors and health disparities further influence pain experiences and treatment outcomes. A multidisciplinary, individualized approach integrating geriatric and SCD-specific principles is critical to improving quality of life in this vulnerable population.

## 1 Introduction

Advances in screening, prophylaxis, and disease-modifying therapies have improved survival among individuals with SCD, resulting in a rapidly expanding population of older adults living with the condition. Once considered as a pediatric disease with limited life expectancy, SCD now spans the life course, bringing into sharper focus the complex and cumulative burden of chronic complications associated with aging (1). Among these, chronic pain is one of the most pervasive, debilitating, and undertreated sequelae, significantly shaping morbidity, functional status, and quality of life in aging adults with SCD.

Chronic pain in SCD is multifactorial, arising from recurrent vaso-occlusive episodes, progressive organ damage, musculoskeletal degeneration, and neuropathic processes. With advancing age, the nature of pain often shifts from episodic, acute vaso-occlusive crises to more persistent, daily pain states. This transition reflects cumulative tissue injury, central sensitization, and overlapping comorbidities such as avascular necrosis, osteoarthritis, renal insufficiency, and cardiovascular disease. Consequently, the aging SCD population experiences a higher burden of complex, mixed pain syndromes that challenge traditional management paradigms.

The management of chronic pain in older adults with SCD is further complicated by age-related physiological changes, polypharmacy, and increased susceptibility to adverse drug effects. Pharmacologic strategies must balance analgesic efficacy with safety concerns, particularly in the context of declining renal and hepatic function, heightened cardiovascular risk, and altered pharmacokinetics. At the same time, psychosocial factors, including stigma, health care disparities, mental health comorbidities, and barriers to specialty care continue to shape pain experiences and treatment outcomes in this population.

Despite growing recognition of these challenges, there remains a lack of comprehensive, age-specific guidance for the management of chronic pain in older adults with SCD. Much of the existing literature and clinical frameworks are derived from younger populations or from general geriatric pain management, without fully accounting for the unique pathophysiology and lived experiences of individuals aging with SCD. As a result, clinicians often face uncertainty in tailoring therapeutic approaches that address both disease-specific mechanisms and age-related vulnerabilities.

This manuscript seeks to address these gaps by synthesizing current evidence on the epidemiology, pathophysiology, and management of chronic pain in the aging SCD population. It examines the evolving burden of pain across the lifespan, highlights considerations in pharmacologic and nonpharmacologic treatment strategies, and explores the broader psychosocial and structural determinants that influence care. By integrating geriatric principles with SCD-specific insights, this work aims to inform a more nuanced, patient-centered approach to managing chronic pain in older adults living with sickle cell disease.

## 2 Research methodology

This manuscript was developed as a narrative review to provide a comprehensive synthesis of chronic pain in aging adults with SCD. A targeted literature search was conducted using databases including PubMed, Web of Science, Cumulative Index to Nursing and Allied Health Literature (CINAHL), and Embase to identify relevant peer-reviewed articles. The search included literature published from January 1, 2003, through May 1, 2026. The search was conducted between May 1, 2026, and July 21, 2026, to identify published studies relevant to the scope of the review. Search terms included combinations of keywords such as: “sickle cell disease”, “aging”, “older adults”, “chronic pain”, “vaso-occlusive pain”, “Nociplastic pain”, “central sensitization”, “comorbidities”, “end-organ damage”, “pain assessment”, “pharmacologic management”, and “non-pharmacologic management”, “pain management”, “neuropathic pain”, “analgesics”, “geriatric pain”, and “health disparities”. Additional publications were identified by reviewing the reference lists of relevant articles, clinical guidelines, and review papers.

The literature search was organized around three primary concepts: (1) sickle cell disease, (2) chronic pain, and (3) aging. Within each concept, synonyms, related keywords, and controlled vocabulary terms, when available, were combined using the Boolean operator OR. The principal concepts were then combined using AND. The core search therefore followed the structure: (sickle cell disease terms) AND (chronic pain terms) AND (aging terms). Literature specifically examining chronic pain among older adults with SCD remains limited; focused supplementary searches were conducted to capture evidence relevant to the principal themes of the review. These searches combined sickle cell disease and pain terms with terms related to pain mechanisms, end-organ damage and comorbidities, pain assessment, pharmacologic treatment, and non-pharmacologic management. Truncation and phrase searching were used where supported by the database, and search syntax was adapted to the subject headings and indexing conventions of each database.

Priority was given to peer-reviewed studies involving aging adults with SCD, clinical guidelines, systematic reviews, and studies addressing pain mechanisms, comorbidity, aging, functional outcomes, and pain management. Articles were selected based on relevance to the epidemiology, pathophysiology, clinical presentation, and management of chronic pain in SCD, with emphasis on studies addressing adult and aging populations. Foundational studies, recent reviews, clinical guidelines, and key primary research articles were prioritized. Foundational status did not depend solely on publication age, citation frequency, author prominence, or journal impact factor. These studies were used primarily to explain the historical development of the field. Additional sources were identified through reference list review and literature in geriatric pain management and hematology.

Findings were synthesized thematically rather than quantitatively. Within each theme, the review first described the development of the relevant concept, when necessary, and then emphasized current evidence concerning its clinical or translational implications. Areas of convergence, uncertainty, and inconsistency were identified. Conclusions were calibrated to the directness and methodological strength of the available evidence, particularly when SCD-specific data from older adults were limited.

Based on this narrative design, the search was not intended to identify every eligible publication, and formal duplicate screening, risk-of-bias assessment, and quantitative synthesis were not performed. The evidence was synthesized narratively and organized according to the manuscript's principal themes: chronic pain mechanisms, age-related and SCD-associated comorbidities, clinical assessment, and multimodal management.

### 2.1 Eligibility and evidence prioritization

Publications were eligible for inclusion when they met one or more of the following criteria: (1) examined chronic or persistent pain among adults with SCD; (2) addressed nociceptive, neuropathic, or nociplastic pain or peripheral or central sensitization in SCD; (3) examined the relationship of aging, cumulative end-organ damage, or comorbidities to chronic pain in adults with SCD; (4) evaluated pharmacologic, behavioral, rehabilitative, or integrative approaches relevant to chronic SCD pain; or (5) provided clinical guidelines or foundational evidence directly applicable to the assessment or management of chronic pain in aging adults with SCD.

Priority was given to studies involving adults with SCD, particularly middle-aged and older adults; clinical practice guidelines; systematic reviews and meta-analyses; randomized or prospective studies; well-designed observational studies; and mechanistic studies directly related to SCD pain. Because relatively few studies have focused specifically on older adults with SCD, evidence from broader adult SCD populations was included when it informed the mechanisms, assessment, or management of chronic pain across the adult life course. Evidence from non-SCD older-adult chronic pain populations was included selectively when it addressed age-related treatment safety, polypharmacy, functional assessment, or non-pharmacologic management and when comparable SCD-specific evidence was unavailable. The indirect nature of such evidence was identified in the narrative synthesis.

Publications were excluded if they: (1) did not address SCD or a clearly applicable chronic pain or aging concept; (2) focused exclusively on pediatric populations without clear relevance to adult or life-course care; (3) addressed only acute vaso-occlusive pain without implications for persistent or chronic pain; (4) focused solely on laboratory or animal findings without a clear connection to the clinical aims of the review; (5) lacked sufficient information to evaluate the methods or findings; or (6) were editorials, letters, commentaries, conference abstracts, or unsupported expert opinion without substantive empirical or guideline-based evidence. Non-English-language publications were excluded.

### 2.2 Defining aging in sickle cell disease

Aging in SCD should not be defined by chronological age alone. Adults with the same chronological age may differ substantially in cumulative vaso-occlusive exposure, hemolytic burden, end-organ damage, physical function, cognitive status, and physiological reserve. Accordingly, aging in SCD can be considered across at least four overlapping dimensions: chronological age, cumulative disease exposure, biological aging, and functional aging or multimorbidity (2–4).

Chronological age captures the duration of exposure to SCD but does not fully reflect disease burden. Cumulative disease exposure encompasses recurrent vaso-occlusion, chronic hemolysis, anemia, inflammation, endothelial dysfunction, hypoxia, ischemia-reperfusion injury, treatment exposure, and progressive organ damage. Biological aging refers to molecular and cellular changes that may occur earlier or progress more rapidly than expected from chronological age. Emerging epigenetic studies have reported evidence consistent with accelerated biological aging in adults with SCD, although these studies remain relatively small and have not established whether epigenetic-age acceleration directly contributes to chronic pain (2, 3).

Functional aging refers to changes in mobility, strength, endurance, cognition, independence, and physiological reserve. These domains may be influenced by osteonecrosis, stroke, cardiopulmonary disease, kidney disease, chronic anemia, sleep disturbance, depression, and repeated hospitalization. For this reason, an aging-focused review of chronic SCD pain must examine not only whether pain intensity changes with chronological age but also how cumulative injury, multimorbidity, and declining reserve change the mechanisms and consequences of pain.

### 2.3 Pathophysiology of chronic pain in the aging SCD population

It is known that older adults with SCD are more likely to experience chronic pain (4, 5). However, current evidence does not identify a pain mechanism that is unique to older adults with SCD. Instead, aging appears to modify the relative contribution, persistence, and clinical expression of nociceptive, neuropathic, and nociplastic mechanisms. These changes reflect the interplays between lifelong SCD pathophysiology and cumulative tissue injury, age-associated disease, multimorbidity, and reduced physiological reserve (4, 6–9).

Chronic pain in sickle cell disease (SCD), defined as pain persisting for longer than three months, affects approximately 40% of adults and becomes more prevalent with advancing age (10–12). SCD has been characterized as a disease of accelerated aging, with cellular, organ and physical manifestations at a younger age similar to what is seen in elderly individuals in the general population (13). Most adult patients in their third decade reach a stage when chronic organ complications (avascular necrosis, renal failure, retinopathy, pulmonary hypertension) become primary drivers of morbidity and mortality (14). A cohort study found that 62% of adult patients with SCD developed new organ complications with progressive increases in retinopathy (39.7%–53.8%), renal failure (6.7%—23.4%), and avascular necrosis (12.5%—20.4%) (15). Findings from the Pain in Sickle Cell Epidemiology Study (PiSCES) indicate that adults experience pain at home on more than half of observed days, underscoring the persistent nature of pain in this population (13). The progression from intermittent, acute vaso-occlusive episodes to chronic pain reflects the interplay of multiple biological mechanisms. Chronic pain in this population includes nociceptive, neuropathic, and centralized mechanisms, with its frequency and severity tending to increase with advancing age (11).

### 2.4 Mechanistic classification of chronic pain in SCD

Chronic pain in SCD can be conceptualized as arising from three overlapping mechanisms: nociceptive, neuropathic, and nociplastic pain. These categories are clinically useful but should not be viewed as mutually exclusive, because individual patients may exhibit features of more than one mechanism, and the predominant mechanism may change over the course of life. Indeed, recurrent acute vaso-occlusive pain, cumulative tissue damage, peripheral nerve injury, and altered central pain processing may interact to produce a mixed chronic pain phenotype.

Nociceptive pain results from actual or threatened injury to non-neural tissue with activation of peripheral nociceptors. In SCD, recurrent microvascular occlusion, tissue hypoxia, ischemia-reperfusion injury, inflammation, and progressive organ damage provide repeated nociceptive input. In aging adults, persistent nociceptive generators may include avascular necrosis, osteoarthritis, joint collapse, vertebral compression, bone infarction, leg ulcers, and other musculoskeletal or end-organ complications. Pain may be localized to an identifiable site and may worsen with movement, weight-bearing, inflammation, or recurrent vaso-occlusion. Repeated nociceptive input may also promote peripheral sensitization and initiate longer-term changes in central pain processing (7, 16). Consequently, the pain phenotype may shift from predominantly episodic vaso-occlusive pain toward a combination of acute episodes and persistent localized pain. Pain caused by established osteonecrosis or degenerative joint disease is more likely to be mechanically provoked, associated with reduced range of motion, and present between vaso-occlusive episodes (4, 6, 17).

Aging may also introduce pain generators that are not specific to SCD, such as primary osteoarthritis, spinal degeneration, osteoporosis-related injury, or other musculoskeletal disease. In an older adult with SCD, these conditions may coexist with ischemic bone injury and cannot be distinguished by pain intensity alone. The clinically relevant aging effect is therefore not the emergence of a new SCD-specific nociceptive mechanism, but an increased number and complexity of persistent nociceptive sources.

Neuropathic pain arises from a lesion or disease affecting the somatosensory nervous system. Proposed contributors in SCD include ischemic, inflammatory, or compressive injury to the peripheral or central nervous system. Over the lifespan, cumulative exposure to stroke, silent cerebral infarctions, chronic ulcers, avascular necrosis, surgery, diabetes, or spinal disease may increase the number of potential neural injuries. Older adults may consequently experience burning, shooting, electric shock-like pain, tingling, numbness, allodynia, superimposed on nociceptive and vaso-occlusive pain (9, 18). Repeated vaso-occlusive episodes and sustained nociceptive or neuropathic input may lower pain thresholds, increase temporal summation, impair endogenous pain inhibition, and produce hyperalgesia, allodynia, or pain extending beyond the site of the original tissue injury.

Studies using neuropathic pain screening instruments demonstrate that these features occur in a clinically important subgroup of individuals with SCD and may become more common with increasing age (19–21). Nevertheless, neuropathic pain is likely underrecognized because it frequently coexists with nociceptive pain and cannot be reliably identified from pain intensity alone (9, 19).

Nociplastic pain refers to pain associated with altered nociceptive processing that is not fully explained by ongoing tissue injury or a discrete lesion of the somatosensory system. Central sensitization, a state of increased responsiveness within the central nervous system (CNS), is an important mechanism contributing to this phenotype. Repeated nociceptive and neuropathic input may promote peripheral sensitization and increased CNS responsiveness. Quantitative sensory testing studies demonstrate thermal, mechanical, and pressure hypersensitivity in subsets of adults with SCD, supporting altered pain processing (7, 8). With increasing disease duration, persistent input from recurrent vaso-occlusion, damaged joints, ulcers, and peripheral nerves may provide more opportunities for sensitization to develop and become self-sustaining.

Features suggestive of a nociplastic component include widespread or multifocal pain, pain that is disproportionate to identifiable tissue injury, heightened sensory sensitivity, and accompanying fatigue, sleep disturbance, cognitive symptoms, or psychological distress. Quantitative sensory testing studies have identified enhanced sensitivity to thermal, mechanical, and pressure stimuli in subsets of patients with SCD, supporting the presence of altered peripheral and central pain processing (8, 22, 23). These features should not be interpreted as indicating that the pain is psychogenic; rather, they reflect neurobiological alterations in pain amplification and modulation (8, 22–24).

These mechanisms may reinforce one another. Recurrent vaso-occlusion and inflammation produce tissue injury and nociceptive pain; prolonged inflammatory and ischemic exposures may sensitize peripheral nociceptors or damage peripheral nerves; and persistent nociceptive and neuropathic signaling may promote central sensitization and maintain pain between vaso-occlusive episodes. With aging, cumulative organ injury, musculoskeletal degeneration, sleep disruption, psychosocial stress, and repeated treatment exposures may further amplify these pathways. Chronic opioid exposure may also contribute to altered pain processing in some patients, although opioid-induced hyperalgesia cannot be inferred from opioid use or escalating analgesic requirements alone (7, 8, 25). Consequently, chronic SCD pain is often best characterized as a dynamic, mixed-mechanism condition rather than as a single disease process.

Although nociceptive, neuropathic, and nociplastic mechanisms provide a useful framework for classifying chronic SCD pain, the expression of these mechanisms in aging adults is strongly influenced by cumulative end-organ damage and the increasing burden of SCD-related and age-associated comorbidities.

Nevertheless, direct evidence that central sensitization progressively worsens with chronological age in SCD is limited. General aging research suggests that pain modulation, sleep, cognition, and neural recovery may change with age, but these findings cannot be assumed to apply directly to older adults with SCD. The more defensible interpretation is that cumulative pain exposure and multisystem disease may interact with age-related changes in pain modulation, rather than that aging alone causes central sensitization.

### 2.5 Biological aging, inflammation, and vascular dysfunction

SCD is characterized by chronic hemolysis, oxidative stress, endothelial activation, inflammation, hypoxia, and ischemia-reperfusion injury—processes that overlap conceptually with several biological pathways implicated in aging. Recent studies using DNA methylation-based measures have reported accelerated biological aging among adults with SCD (2, 3). These findings provide a potential biological link among cumulative disease stress, organ dysfunction, and declining resilience.

However, evidence directly connecting epigenetic-age acceleration, cellular senescence, or “inflammaging” to chronic pain in SCD is not yet sufficient to establish causality. It remains unknown whether biological-age acceleration precedes chronic pain, results from disease burden and treatment exposure, or shares common upstream mechanisms with pain. These pathways should therefore be presented as an emerging research framework rather than as established mechanisms specific to older adults with SCD.

Collectively, these mechanisms support the characterization of SCD as a condition of accelerated aging, marked by progressive cellular and organ dysfunction. Clinical manifestations, including stroke, cardiomegaly, heart failure, pulmonary hypertension, nephropathy, osteopenia, osteoporosis, osteonecrosis, and gout, mirror age-related conditions seen in the general population, further compounding the complexity of chronic pain in aging individuals with SCD.

### 2.6 Reduced reserve and pain persistence

Aging-related reductions in physiological reserve may affect the consequences and recovery trajectory of pain even when the initiating mechanism is unchanged. Cardiopulmonary disease, chronic kidney disease, anemia, muscle weakness, cognitive impairment, and poor sleep may reduce mobility and delay recovery after a painful episode. Pain-related activity avoidance can then contribute to deconditioning, stiffness, reduced strength, and increased pain interference, creating a reciprocal cycle among pain, disability, and declining reserve.

This distinction is important: aging may not always increase the intensity of the original nociceptive signal, but it may increase pain duration, functional consequences, treatment burden, and the time required to return to baseline.

### 2.7 End-organ damage, age-related comorbidities, and chronic pain

As individuals with SCD survive into middle and older adulthood, chronic pain increasingly reflects the combined effects of recurrent vaso-occlusion, cumulative end-organ damage, age-related disease, and altered pain processing. Acute vaso-occlusive pain generally results from episodic microvascular obstruction, ischemia-reperfusion injury, inflammation, and tissue hypoxia. It often has a sudden onset, may involve the back, chest, abdomen, or extremities, and may resolve as the acute episode subsides. In contrast, pain related to established organ damage or an age-associated comorbidity is more likely to be persistent, anatomically localized, mechanically provoked, or associated with functional impairment. Nevertheless, the distinction is not always clear because an acute vaso-occlusive episode can occur at the site of preexisting tissue damage, and chronic structural pathology can predispose patients to recurrent pain exacerbations (6, 7, 10, 26).

Musculoskeletal complications are among the most important persistent pain generators in adults with SCD. Repeated bone and marrow ischemia can cause osteonecrosis, particularly in the femoral or humeral head, which may progress to joint collapse and secondary degenerative arthritis. Unlike an acute vaso-occlusive episode, osteonecrosis commonly produces localized hip or shoulder pain that is aggravated by movement or weight-bearing and accompanied by reduced range of motion and progressive functional limitation. Vertebral infarction, compression deformities, chronic bone injury, osteomyelitis, and age-related osteoarthritis may produce additional nociceptive and mechanical pain. Because these disorders can coexist with vaso-occlusive pain, new, localized, progressively worsening, or function-limiting pain should not automatically be attributed to an acute pain episode (7, 17, 26).

Chronic leg ulcers represent another distinct source of persistent pain. Tissue ischemia, inflammation, edema, infection, and repeated disruption of the wound can generate ongoing nociceptive pain, while local nerve injury may produce burning, tingling, allodynia, or other neuropathic features. Leg-ulcer pain may therefore persist between vaso-occlusive episodes and contribute to impaired mobility, sleep disruption, social isolation, and reduced quality of life. The association of leg ulcers with other vasculopathic complications, including renal and cardiopulmonary disease, further illustrates how chronic pain and end-organ damage may cluster rather than occur independently (27, 28).

Neurologic and cerebrovascular complications may also alter the pain phenotype. Stroke or peripheral nerve injury can produce neuropathic pain characterized by burning, shooting, electric shock-like sensations, numbness, or pain within a neuroanatomic distribution. Cognitive impairment following overt or silent cerebral injury may make it more difficult for older adults to characterize pain, follow complex medication regimens, or use behavioral self-management strategies. These factors can complicate the differentiation of acute vaso-occlusive pain from chronic neuropathic or centralized pain and may increase reliance on caregivers and health services (9, 10).

Some end-organ complications may influence pain indirectly rather than serve as the primary pain generator. Chronic kidney disease, hepatic dysfunction, pulmonary hypertension, cardiopulmonary disease, and reduced exercise capacity may increase fatigue, disrupt sleep, restrict mobility, and contribute to physical deconditioning. Reduced activity can subsequently worsen joint stiffness, muscle weakness, disability, and pain interference. These conditions also narrow the therapeutic window: renal, hepatic, cardiopulmonary, and cognitive vulnerabilities may affect the selection, dosage, and monitoring of nonsteroidal anti-inflammatory drugs, opioids, and adjuvant analgesics. Thus, a comorbidity may increase the burden of pain while simultaneously limiting treatment options (6, 10, 11).

Common age-related conditions, including osteoarthritis, obesity, diabetes, osteoporosis, and spinal degenerative disease, should also be evaluated as potential pain contributors. Their presence does not mean that the patient's SCD-related pain is less valid. Instead, these conditions can introduce additional nociceptive, inflammatory, mechanical, or neuropathic mechanisms. Sleep disturbance, depression, anxiety, and psychosocial stress may further amplify pain intensity and interference through bidirectional biological and behavioral pathways. These factors are best understood as clinically relevant pain modifiers rather than evidence that the pain is primarily psychological (18, 29–31).

Importantly, vaso-occlusion, organ damage, and age-related comorbidities are not independent pathways. Repeated vaso-occlusion can cause cumulative bone, joint, organ, and nerve injury. Persistent nociceptive and neuropathic input from these damaged tissues may subsequently promote peripheral sensitization and central amplification, allowing pain to persist after the initiating injury or acute episode has resolved. Chronic pain in aging adults with SCD is therefore frequently a mixed-mechanism condition involving episodic vaso-occlusive pain superimposed on one or more persistent nociceptive, neuropathic, or nociplastic pain processes (6, 7, 30).

### 2.8 Guidelines

The 2020 guidelines from the American Society of Hematology (ASH) advocate for a multimodal strategy in managing chronic pain associated with sickle cell disease (SCD), emphasizing that pharmacologic treatment alone is often insufficient to adequately address both the physical and psychosocial dimensions of chronic pain (11). The key recommendations include non-pharmacologic strategies in addition to first-line pharmacologic management.

Nonpharmacologic strategies should be integrated with medication-based treatment. These approaches incorporate psychological interventions such as cognitive behavioral therapy (CBT), mindfulness practices, acceptance and commitment therapy (ACT), and coping skills training, as well as physical modalities like exercise and yoga. Integrative therapies, including massage, acupuncture, music therapy, and other complementary approaches, may also support comprehensive pain management (11).

First-line pharmacologic management typically includes nonsteroidal anti-inflammatory drugs (NSAIDs) and opioid analgesics. The panel underscores the importance of prioritizing patient-centered outcomes, including pain severity, coping mechanisms, reductions in chronic opioid use (measured as daily morphine milligram equivalents), health care utilization, health-related quality of life, functional status, sleep quality, and emotional well-being (11).

### 2.9 Pharmacological management

#### 2.9.1 Age-informed pharmacologic management

Pharmacologic management of chronic pain in aging adults with SCD requires a distinction between medications that directly treat pain and disease-modifying therapies intended to reduce vaso-occlusion or other SCD complications. Hydroxyurea, L-glutamine, and crizanlizumab may reduce vaso-occlusive events in patients with SCD but should not be described as direct treatments for all forms of established chronic pain. Persistent pain caused by osteonecrosis, neuropathic injury, degenerative joint disease, chronic ulcers, or altered nociceptive processing may continue despite a reduction in acute vaso-occlusive episodes (4, 11).

Evidence to guide pharmacologic treatment specifically in adults over sixty with SCD is limited. Consequently, treatment requires integration of SCD-specific pain guidance with geriatric prescribing principles. Decisions should be based on the pain mechanism, organ function, frailty, cognitive and functional status, concurrent medications, treatment history, and patient goals rather than chronological age alone (11, 32–35).

#### 2.9.2 Pharmacokinetic and pharmacodynamic changes

Aging can alter drug absorption, distribution, metabolism, and elimination. Reductions in total body water and lean body mass may increase concentrations of water-soluble medications, while increased body fat may prolong the effects of lipophilic drugs. Reduced hepatic blood flow and metabolic capacity can lengthen the duration of some medications, and declining renal clearance can cause accumulation of parent drugs or active metabolites. These changes may be amplified in SCD by chronic kidney disease, hepatic injury, cardiopulmonary disease, low body weight, malnutrition, or cumulative treatment exposure (32–34).

Older or frail adults may also demonstrate increased pharmacodynamic sensitivity to opioids, sedatives, anticholinergic medications, and other centrally acting drugs (35). A dose tolerated in a younger adult may cause sedation, confusion, impaired balance, or respiratory suppression in an older patient even when serum drug concentrations are similar. Medication initiation should therefore generally involve conservative dosing and careful titration, while recognizing that cautious prescribing should not become therapeutic inertia or undertreatment (32–34, 36).

The NHLBI Expert Panel advises clinicians to first identify the underlying cause and classification of chronic pain, such as avascular necrosis, leg ulcers, or pain without clear objective findings potentially related to neuroplastic changes (37). They recommend using patient response to therapy to inform decisions regarding long-term opioid use and emphasize the importance of developing a written, individualized pain management plan supported by a patient-provider partnership agreement. Key pharmacological considerations include opioids, adjuvant agents, NSAIDS, in the setting of disease modifiers.

#### 2.9.3 Non-opioid analgesics

Acetaminophen may be useful for localized nociceptive or musculoskeletal pain, but the total daily exposure should be reviewed across prescription and nonprescription combination products. Low body weight, poor nutrition, regular alcohol use, and hepatic dysfunction may reduce the margin of safety. Acetaminophen may be incorporated into multimodal care but is unlikely to control severe mixed-mechanism SCD pain when used alone (32–34).

Nonsteroidal anti-inflammatory drugs may provide short-term benefit for inflammatory or musculoskeletal pain, but their geriatric risk profile is particularly relevant in SCD. Older adults are more susceptible to gastrointestinal bleeding, acute kidney injury, fluid retention, and cardiovascular adverse effects. These concerns are magnified by SCD nephropathy, hypertension, anticoagulant or antiplatelet treatment, and preexisting cardiopulmonary disease (32, 36).

The ASH 2020 guidelines indicate that nonsteroidal anti-inflammatory drugs (NSAIDs), including both selective and nonselective cyclooxygenase (COX) inhibitors, may be used as adjunctive therapy with opioids for the management of acute pain, although this recommendation is based on very low-certainty evidence. Careful, individualized evaluation of renal, cardiovascular, and gastrointestinal risks is essential when considering their use. Both selective COX-2 inhibitors and nonselective NSAIDs have been associated with increased risks of kidney disease and cerebrovascular events, conditions that are already highly prevalent among adults with SCD. Notably, ketorolac has been specifically linked to an elevated risk of acute kidney injury in this population, due to the high prevalence of sickle cell-related kidney disease, with microalbuminuria affecting approximately 30% of adults and progression to end-stage renal disease observed in up to 25% of older individuals (10, 11).

Renal function, gastrointestinal bleeding history, cardiovascular status, hydration, and interacting medications should be reviewed before treatment. Repeated or prolonged NSAID use should be avoided or approached cautiously in patients with chronic kidney disease or high bleeding risk. The balance differs from that in a younger adult with more preserved renal function and no interacting medications.

#### 2.9.4 Opioid therapy

Opioid analgesics continue to play a central role in the management of chronic SCD-related pain that is not adequately controlled with nonopioid therapies. Both long-acting and short-acting formulations may be utilized as clinically indicated, ideally coordinated by a single prescribing clinician who provides prescriptions every two weeks to one month, along with in-person assessments at intervals of approximately 2–3 months (37). Although concerns about opioid use disorder persist, available evidence suggests that its prevalence in the SCD population has remained relatively low, with stable prescribing patterns observed between 2009 and 2014 (38). Particularly, individuals with higher levels of depressive symptoms may be at greater risk for opioid misuse, highlighting the importance of comprehensive psychosocial assessment (11, 13, 14).

Opioids may remain necessary for some adults with severe acute or chronic SCD pain. Aging does not eliminate the ethical obligation to treat pain adequately, and chronological age alone should not preclude opioid treatment. However, older adults may be more susceptible to sedation, constipation, respiratory depression, delirium, falls, and fractures. A meta-analysis found an association between opioid use and falls, fall-related injuries, and fractures among older adults, although the consequences of inadequately treated pain must also be considered (34, 36, 39, 40).

Renal and hepatic function influence opioid selection because several opioids or their metabolites can accumulate when clearance is impaired. Medication choice should therefore be individualized rather than based on a single preferred opioid for all older adults. Assessment should include kidney and liver function, sleep apnea or pulmonary disease, prior opioid exposure, cognitive status, fall history, constipation, and the use of other central nervous system depressants.

Particular caution is warranted when opioids are combined with benzodiazepines, gabapentinoids, sedating antihistamines, muscle relaxants, alcohol, or cannabinoids. Such combinations may increase sedation, cognitive impairment, respiratory suppression, and falls. The 2023 American Geriatrics Society Beers Criteria recommends avoiding several high-risk combinations in older adults, although these criteria should support individualized review rather than automatic discontinuation of clinically necessary treatment (36).

For an older or frail adult initiating opioid therapy, a lower starting dose and slower titration may be appropriate. For an opioid-tolerant patient, however, empiric dose reduction based solely on age can precipitate withdrawal or uncontrolled pain. Established therapy should be reassessed collaboratively, and any reduction should be individualized and gradual. Monitoring should include pain relief, function, alertness, cognition, gait, falls, respiratory symptoms, bowel function, and adherence. Naloxone education and access should be considered as part of opioid safety planning.

#### 2.9.5 Medications for neuropathic pain

Adjuvant therapies are recommended to address neuropathic components of pain and may include antidepressants (such as duloxetine and amitriptyline), anticonvulsants (including gabapentin and pregabalin), and topical agents like lidocaine (41).

Gabapentinoids, serotonin-norepinephrine reuptake inhibitors (SNRIs), and selected antidepressants may be considered when neuropathic features are present, but their risks become more consequential with aging.

Gabapentin and pregabalin require adjustment for renal function and may cause dizziness, somnolence, gait instability, edema, or cognitive slowing. Their concurrent use with opioids may increase sedation and respiratory risk. Serotonin-norepinephrine reuptake inhibitors may be useful for neuropathic pain with co-occurring depression or anxiety, but treatment should consider renal or hepatic impairment, blood pressure, hyponatremia risk, and interactions with other serotonergic medications.

Tricyclic antidepressants carry anticholinergic and cardiovascular risks, including confusion, constipation, urinary retention, orthostatic hypotension, and falls. These effects may outweigh potential benefit in a frail adult or someone with cognitive impairment. Medication selection should therefore be guided by the patient's full geriatric and comorbidity profile rather than by the neuropathic-pain indication alone (36, 42).

#### 2.9.6 Cannabinoids

The role of cannabis in the management of chronic pain in sickle cell disease (SCD) remains uncertain (43, 44). Evidence from a small randomized, placebo-controlled crossover trial involving 23 participants found no significant difference in mean pain scores over a five-day period with the use of vaporized cannabis (11).

Preclinical studies, however, suggest that purified cannabidiol (CBD) may offer potential analgesic and disease-modifying effects (45, 46). These benefits are thought to occur through modulation of pro-inflammatory immune responses, reduction of oxidative stress, and attenuation of neurogenic inflammation (47). Despite these promising mechanisms, clinical data in humans are limited, and further research is needed to clarify the safety, efficacy, and role of cannabis-based therapies in the management of chronic SCD pain. A small, randomized trial of vaporized cannabis in adults with SCD demonstrated feasibility and potential effects on some pain-related outcomes, but it did not establish long-term safety or effectiveness in older or frail adults. Older patients may be more vulnerable to dizziness, orthostatic symptoms, sedation, altered attention, impaired balance, and interactions with opioids or other central nervous system depressants. Product composition and tetrahydrocannabinol exposure may also be inconsistent.

Cannabinoids should therefore not be presented as established geriatric SCD pain therapy. If considered, clinicians should evaluate cognition, fall risk, cardiovascular disease, psychiatric history, concurrent sedatives, route of administration, and local regulatory requirements. Evidence specific to adults over sixty with SCD is lacking.

#### 2.9.7 Disease-modifying SCD therapy

Disease-modifying therapy remains important because reducing vaso-occlusive events and cumulative organ injury may reduce one source of pain. However, disease modification and chronic-pain treatment are not synonymous.

Hydroxyurea is considered a first-line disease-modifying therapy for sickle cell disease, associated with a 44% reduction in vaso-occlusive crises and a 40% reduction in mortality over long-term follow-up (48, 49). Hydroxyurea may reduce vaso-occlusive events and acute-care utilization but requires monitoring of blood counts, adherence, renal function, and treatment tolerance. In older adults, marrow reserve, kidney function, multimorbidity, and the feasibility of laboratory monitoring should inform dosing and follow-up. Age alone should not be a reason to withhold hydroxyurea, but evidence specific to adults over sixty remains limited. Despite its demonstrated benefits, utilization remains suboptimal, with only approximately 23% of eligible adults filling prescriptions (11).

Voxelotor (Oxbryta) has been voluntarily withdrawn from clinical use following reports of increased rates of vaso-occlusive crises and mortality. In contrast, crizanlizumab (Adakveo) and L-glutamine (Endari) remain approved by the U.S. Food and Drug Administration for the prevention of vaso-occlusive episodes (11). It should however be noted that these drugs have been studied in the context of acute pain crises and not chronic pain.

Crizanlizumab is intended to reduce vaso-occlusive crises rather than directly treat neuropathic, degenerative, or nociplastic pain. Trials generally enrolled adolescents and adults but provided limited evidence specific to the oldest adults with SCD. Decisions should consider the patient's vaso-occlusive phenotype, infusion burden, comorbidity, adverse reactions, current regulatory status, availability, and the evolving evidence base (50).

#### 2.9.8 Polypharmacy and frailty

A structured medication review should identify therapeutic duplication, medications without a current indication, anticholinergic burden, CNS depressants, serotonergic combinations, nephrotoxic medications, and drugs that increase orthostatic or bleeding risk. Medication reconciliation should include prescriptions from multiple clinicians, over-the-counter products, supplements, alcohol, and cannabis.

Frailty may be more informative than chronological age alone. Weight loss, weakness, slow gait, exhaustion, falls, impaired instrumental activities, cognitive change, and limited social support may affect the ability to tolerate or safely self-administer complex regimens. Simplifying dosing schedules, using written medication plans, involving a pharmacist, and including caregivers with the patient's permission may improve safety.

### 2.10 Non-pharmacologic management

Nonpharmacological strategies are essential components of comprehensive chronic pain management in aging adults with sickle cell disease (SCD) and should be used alongside pharmacologic therapies (11, 51). They are not substitutes for disease-modifying treatment, evaluation of new or worsening complications, or appropriately indicated analgesic therapy. Rather, these interventions address dimensions of chronic pain that medication alone may not fully improve, including mobility, physical deconditioning, sleep, coping, emotional distress, self-efficacy, participation in daily activities, and quality of life. These approaches target physical, psychological, and behavioral dimensions of pain, helping to improve function and overall quality of life.

#### 2.10.1 Cognitive-behavioral therapy

The rationale for integrating these strategies may be particularly compelling in aging adults with SCD. Age-related physiological changes, cumulative organ damage, multimorbidity, and polypharmacy may alter medication metabolism and increase susceptibility to sedation, cognitive impairment, falls, respiratory depression, gastrointestinal bleeding, renal injury, and drug–drug interactions. Renal, hepatic, cardiopulmonary, and neurologic complications may further narrow the therapeutic window for opioids, nonsteroidal anti-inflammatory drugs, and adjuvant analgesics. Non-pharmacologic approaches may help improve function and coping without adding to the pharmacologic burden, although they should not be presented primarily as a means of withholding or discontinuing necessary analgesics (11, 32, 33).

The American Society of Hematology conditionally recommends cognitive and behavioral pain-management strategies for chronic SCD pain, while emphasizing that the certainty of the available evidence is generally low (11). The NHLBI guidelines support the use of nonpharmacological interventions such as deep tissue massage, progressive muscle relaxation, and self-hypnosis, although these recommendations are based on limited and low quality evidence (11, 13). Additional strategies include cognitive behavioral therapy (CBT), occupational therapy, acupuncture, and engagement in low- to moderate-intensity physical activity, including aquatic therapy (11, 13). In a multicenter randomized controlled trial, a digital CBT program for adolescents with SCD was associated with moderate improvements in average pain intensity at six months (effect size d = 0.50) and a reduction in the number of pain days (incidence rate ratio [IRR] = 0.63, *p* = 0.006) (52). Findings from a Cochrane review further suggest that CBT may improve the emotional aspects of pain, although it does not appear to significantly reduce pain intensity overall, and the broader evidence base for psychological interventions in SCD remains limited (47).

Rehabilitative strategies such as physical therapy, occupational therapy, and structured exercise programs including low- to moderate-intensity activities and aquatic therapy can improve mobility, reduce deconditioning, and support functional independence. Integrative therapies, including massage, acupuncture, and relaxation techniques (e.g., guided imagery, progressive muscle relaxation, and self-hypnosis), are also recommended, although the supporting evidence remains limited (11).

#### 2.10.2 Physical therapy, occupational therapy, and graded activity

Physical therapy can address reduced range of motion, muscle weakness, joint instability, gait abnormalities, and deconditioning associated with chronic pain, osteonecrosis, degenerative joint disease, prior stroke, or prolonged activity avoidance. Treatment may include individualized stretching, strengthening, mobility exercises, posture and gait training, and aerobic conditioning. Occupational therapy can support energy conservation, joint protection, activity pacing, adaptive equipment, and modifications that facilitate independence in work, household tasks, and self-care.

For older adults, activity plans should be individualized according to cardiopulmonary status, anemia, balance, bone and joint disease, fall risk, and baseline functional capacity. Gradual progression is preferable to abrupt increases in activity. Appropriate hydration, avoidance of temperature extremes, and attention to symptoms such as chest pain, marked dyspnea, dizziness, or unusual fatigue are important. The therapeutic goal is not simply increased exercise but improved function, confidence, and participation without causing sustained pain exacerbation. Although exercise and rehabilitation are biologically and functionally plausible interventions, controlled evidence establishing their effects on chronic SCD pain remains limited (11, 32, 33, 53).

#### 2.10.3 Mindfulness, relaxation, and stress-management approaches

Mindfulness-based interventions encourage nonjudgmental awareness of pain, thoughts, emotions, and bodily sensations. Relaxation strategies may include diaphragmatic breathing, guided imagery, progressive muscle relaxation, and meditation. These approaches may reduce autonomic arousal, stress reactivity, and pain-related distress and may help patients respond to pain with less fear and behavioral disruption. A pilot study in adults with SCD and chronic pain demonstrated the feasibility and acceptability of a mindfulness-based intervention, but larger controlled trials are needed to determine its effectiveness and durability (54).

These practices may be adapted for an older adult's cognitive ability, sensory limitations, cultural preferences, and prior experience. Brief, repeated practices may be more feasible than lengthy sessions for individuals with fatigue or limited concentration.

#### 2.10.4 Sleep-focused interventions

Sleep disturbance and chronic pain can reinforce one another. Pain may interfere with sleep initiation and maintenance, while insufficient or fragmented sleep may heighten pain sensitivity, fatigue, mood symptoms, and functional impairment. Screening should address insomnia, sleep apnea, restless legs symptoms, nocturnal hypoxemia, and medication-related sedation.

Non-pharmacologic approaches may include sleep education, consistent sleep and wake times, stimulus control, relaxation, and cognitive-behavioral therapy for insomnia. Treating an underlying sleep disorder is particularly important before escalating sedating medications. SCD-specific intervention evidence is limited, but sleep assessment is clinically relevant within comprehensive chronic pain care (32, 33).

#### 2.10.5 Massage and other manual approaches

Massage may reduce muscle tension, promote relaxation, and provide short-term relief for some patients. A small randomized trial in younger patients with SCD found potential short-term benefits, but the findings cannot be assumed to apply directly to older adults (55). Massage should be individualized and avoided or modified over acute swelling, open leg ulcers, suspected infection, unstable joints, severe osteonecrosis, or areas of acute tissue injury. It should be presented as an optional adjunct guided by patient preference rather than an established treatment for the underlying SCD pathology.

#### 2.10.6 Acupuncture

Acupuncture has been used as an adjunctive pain treatment in SCD, and small observational studies suggest feasibility and possible short-term benefit (56–58). However, the available studies are limited by small samples, nonrandomized designs, and limited representation of older adults. Acupuncture may be considered for interested patients when delivered by a qualified practitioner and when contraindications, such as infection risk, anticoagulation, skin integrity, and bleeding risk, have been evaluated. Current evidence is insufficient to recommend it as a replacement for established treatment.

#### 2.10.7 Transcutaneous electrical nerve stimulation

Transcutaneous electrical nerve stimulation (TENS) delivers low-voltage electrical stimulation through skin electrodes and may be considered for localized musculoskeletal or neuropathic pain. However, a Cochrane review identified only one small crossover trial in SCD and concluded that the evidence was insufficient to establish benefit or harm (59). Therefore, TENS should be described as an optional, individualized adjunct rather than a proven SCD pain treatment. Placement over impaired skin, ulcers, or areas with reduced sensation should be avoided, and safe use may require instruction from a rehabilitation or pain professional.

Overall, while the quality of evidence varies, these interventions offer low-risk, patient-centered options that address the multidimensional nature of chronic pain in SCD. A tailored, multidisciplinary approach is critical, particularly in older adults with complex comorbidities and functional limitations (11). No single non-pharmacologic intervention is appropriate for every aging adult with SCD. Selection should be guided by the predominant pain mechanism, functional limitations, comorbidities, cognitive and sensory status, patient preferences, treatment burden, transportation, cost, and local availability. Outcomes should extend beyond pain intensity to include physical function, sleep, emotional well-being, participation, self-efficacy, medication-related adverse effects, and quality of life. A multimodal plan should be reassessed periodically and coordinated among hematology, primary care, pain medicine, rehabilitation, behavioral health, and other relevant disciplines.

### 2.11 Psychosocial factors

Psychosocial factors are not peripheral to chronic SCD pain. They influence pain appraisal, coping, healthcare use, treatment adherence, sleep, physical activity, and participation in valued roles. Chronic pain in aging adults with sickle cell disease (SCD) is accompanied by a substantial psychosocial burden that significantly affects overall well-being (60, 61). Depression, anxiety, stigma, discrimination, and mistrust are well documented in the broader adult SCD population (18, 29, 62, 63). The prevalence of depression in individuals with SCD is notably higher than in the general population, approximately threefold (26% vs. 9.5%) and is closely linked to increased pain frequency, greater healthcare utilization, and poorer health-related quality of life (64).

An aging-focused analysis must additionally consider how these experiences interact with retirement, bereavement, shrinking support networks, functional loss, cognitive change, multimorbidity, and increasing treatment burden. Direct evidence describing these processes among adults over sixty with SCD remains limited. Consequently, some aging-specific relationships are supported primarily by broader older-adult or chronic-pain research and should be identified as clinically plausible rather than conclusively established in older adults with SCD.

#### 2.11.1 Depression, anxiety, and cumulative psychological burden

Depression and anxiety are associated with poorer quality of life, greater pain burden, and increased healthcare utilization among adults with SCD (29, 63). With aging, these symptoms may occur in the context of cumulative disease exposure, repeated hospitalization, progressive organ injury, bereavement, functional loss, financial strain, and uncertainty about future independence. The meaning of psychological distress may therefore change across the life course, even if the symptoms themselves are not unique to older age. Elevated depressive symptoms are further associated with higher daily pain intensity, more negative mood, greater pain interference and catastrophizing, diminished quality of life, and an increased risk of opioid misuse (14).

Findings from the international Sickle Cell World Assessment Survey (SWAY) highlight the broad and multidimensional impact of SCD on daily functioning and emotional health (65). In addition to depression, individuals with SCD frequently experience anxiety, post-traumatic stress disorder (PTSD), sleep disturbances, loneliness, feelings of helplessness, and reliance on pain medications (66).

Assessment can be complicated by overlap among depression, chronic pain, fatigue, sleep disturbance, anemia, medication effects, and physical illness. Clinicians should avoid assuming that reduced activity, impaired concentration, or sleep disruption is entirely psychological or entirely attributable to SCD. Screening should be followed by clinical assessment that considers medical, cognitive, functional, and social contributors.

#### 2.11.2 Retirement, work loss, and financial insecurity

Retirement may reduce occupational stress for some adults, but it can also remove daily structure, social contact, income, insurance stability, and a valued source of identity. For individuals with SCD, departure from the workforce may occur earlier than expected because of pain, disability, recurrent hospitalization, or workplace discrimination. Thus, “retirement” may represent involuntary work loss rather than a planned later-life transition.

Reduced income may make transportation, medications, home assistance, nutritious food, and non-pharmacologic pain treatments less accessible. These barriers can increase stress and limit the feasibility of complex care plans. Psychosocial assessment should therefore include employment history, financial strain, insurance, housing, transportation, and the patient's access to disability and community resources.

#### 2.11.3 Social isolation and changing support networks

Chronic pain can restrict mobility and participation in social activities, while social isolation may worsen emotional distress and pain-related disability (67, 68). With aging, retirement, death or illness of family members, relocation of adult children, reduced ability to drive, hearing or visual limitations, and decreased participation in community or faith-based activities may contract the patient's social network.

Social isolation and loneliness are related but distinct. Social isolation reflects limited social contact, whereas loneliness refers to a perceived gap between desired and available connection. A patient may live alone without feeling lonely or live with family while experiencing profound loneliness. Both structural support and the patient's subjective experience should therefore be assessed.

Social isolation does not necessarily increase with age among people with SCD, rather, older adults with chronic pain may face multiple converging risks for reduced social connection.

#### 2.11.4 Caregiver loss and changing family roles

Many adults with SCD have relied on parents, siblings, partners, or close friends during pain episodes and hospitalizations. As patients age, long-standing caregivers may themselves develop illness, disability, or cognitive decline, or may die. The loss of a parent or partner may remove not only emotional support but also someone who understands the patient's baseline pain pattern, medication regimen, communication preferences, and prior experiences with healthcare discrimination.

Family roles may also reverse. An older adult with SCD may become a caregiver for an aging parent, spouse, sibling, or grandchild while simultaneously requiring assistance with their own health needs. Alternatively, adult children or spouses may assume increasing responsibility for transportation, medication management, household activities, or communication with clinicians. These transitions can generate grief, guilt, conflict, and concern about being a burden.

Caregiver involvement should therefore be guided by the patient's preferences and should preserve autonomy wherever possible. Supportive care should include assessment of both available assistance and caregiver strain.

#### 2.11.5 Functional decline, frailty, and increasing dependency

Chronic pain, osteonecrosis, stroke, cardiopulmonary disease, kidney disease, muscle weakness, and fatigue may reduce mobility and independence. Difficulty with bathing, dressing, meal preparation, transportation, or medication management can threaten identity and autonomy, particularly for adults who have spent decades developing strategies to manage SCD independently.

Frailty may intensify the relationship between pain and psychosocial distress. Pain can reduce activity, resulting in deconditioning and greater dependence; increasing dependence may then contribute to social withdrawal, depressed mood, and fear of further decline. These relationships are likely bidirectional rather than linear.

Assessment should include activities of daily living, instrumental activities, mobility, falls, fatigue, cognition, social participation, and the extent to which pain interferes with personally meaningful roles, not merely pain intensity.

#### 2.11.6 Cognitive change and self-advocacy

Cerebrovascular injury and cognitive impairment may affect memory, processing speed, executive function, medication management, appointment attendance, and the ability to communicate a complex pain history (69). These challenges may be mistakenly interpreted as nonadherence, indifference, or drug-seeking behavior. Cognitive changes may also weaken a patient's ability to advocate effectively in clinical encounters where SCD pain is already subject to disbelief or stigma.

Clinicians should use clear communication, medication reconciliation, written treatment plans, teach-back, and supportive involvement of a trusted person when authorized by the patient. Cognitive impairment should not be presumed from age alone, nor should it negate the patient's role in shared decision-making.

#### 2.11.6 Multimorbidity and treatment burden

As adults with SCD age, they may manage several interacting conditions, multiple clinicians, frequent appointments, laboratory monitoring, complex medication schedules, and competing recommendations. Multimorbidity is associated in older populations with disability, poorer function, reduced quality of life, and treatment burden (70, 71). In SCD, fragmented specialty care may require the patient to coordinate hematology, primary care, nephrology, cardiology, neurology, orthopedics, pain management, and behavioral health.

Treatment burden can itself become a psychosocial stressor. Recommendations should therefore be evaluated not only for clinical efficacy but also for feasibility, cumulative workload, transportation requirements, cost, and alignment with the patient's priorities.

#### 2.11.7 Cumulative stigma, discrimination, and ageism

SCD-related stigma and discrimination are well established, particularly during encounters for pain treatment (62). Individuals with SCD are also especially susceptible to stigma and discrimination in clinical settings, as their pain is often not accompanied by observable physical findings (63, 72).

Approximately 60% of individuals with SCD report avoiding emergency department (ED) care due to prior negative experiences. Among those who do seek care, studies indicate an average wait time of about 90 min before receiving analgesic treatment (73). Notably, despite presenting with higher pain scores and greater triage acuity, patients with SCD experience longer delays to initial analgesia compared to individuals with conditions such as renal colic (74). As such, validating and trusting patient-reported pain is essential for effective and patient-centered pain management (13). The psychosocial impact of SCD extends beyond the individual, affecting family members who often experience emotional distress, financial burden, and disruptions in social and daily functioning (66).

Aging may add another layer of vulnerability. Older adults may encounter age-related assumptions about cognition, opioid use, functional potential, or the appropriateness of intensive treatment. They may also experience the cumulative psychological effects of decades of delayed analgesia, disbelief, racial bias, and fragmented care.

The intersection of SCD stigma, racism, disability, and ageism may influence trust and willingness to seek care. However, direct empirical evidence on this intersection in older adults with SCD is limited. These factors underscore the importance of integrating psychosocial assessment and support into comprehensive pain management strategies for aging adults with SCD.

#### 2.11.8 Resilience and accumulated expertise

An aging-focused framework should not portray older adults only through vulnerability and decline. Adults who have lived with SCD for decades may possess substantial knowledge about their pain patterns, triggers, effective treatments, adverse medication effects, and healthcare navigation. Many have developed adaptive coping, advocacy skills, spiritual resources, family strategies, and community relationships that support resilience.

Clinical care should treat this lived expertise as a resource. Shared decision-making should begin by asking what the patient has learned over time, what has previously helped or caused harm, which roles and activities matter most, and what level of treatment burden is acceptable.

### 2.12 Health disparities and barriers to care

Significant health disparities and structural barriers continue to shape the experience and management of chronic pain in individuals with sickle cell disease (SCD), particularly among aging adults (75, 76). SCD disproportionately affects individuals of African descent and other historically marginalized populations, placing patients at the intersection of racial, socioeconomic, and healthcare inequities (12, 65, 77). These disparities contribute to unequal access to high-quality pain management, specialty care, and disease-modifying therapies.

Bias and stigma within healthcare settings remain pervasive and are well-documented contributors to suboptimal pain treatment (78–80). Patients with SCD are frequently perceived as drug-seeking, particularly in acute care environments, which can result in delayed or inadequate analgesia despite high pain acuity (78–81). Such experiences erode trust in the healthcare system and contribute to care avoidance, delays in treatment, and poorer clinical outcomes.

System-level barriers further compound these challenges. Limited access to comprehensive SCD centers, fragmented care during the transition from pediatric to adult services, and inadequate provider training in SCD pain management all contribute to inconsistent and often insufficient care. Insurance-related barriers and restrictive opioid prescribing policies—despite not being intended for SCD populations—have also reduced access to appropriate analgesic therapy.

Social determinants of health, including income instability, transportation challenges, and reduced access to supportive resources, further hinder engagement in care and adherence to treatment plans. For aging adults, these challenges are often magnified by increasing comorbidity, disease burden and functional limitations.

2016 CDC Guideline for Prescribing Opioids for Chronic Pain has also had unintended consequences for this population. Although not intended to apply to SCD, subsequent research published in *JAMA Internal Medicine* found that the guideline was associated with reduced opioid prescribing in SCD patients, thereby creating additional barriers to accessing appropriate pain management (12). While the CDC clarified in 2019 that the guideline should not restrict opioid use in SCD, these clarifications have not been consistently reflected in clinical practice (12).

Provider-related concerns further contribute to treatment barriers. Surveys of clinicians caring for individuals with SCD indicate that fears surrounding drug dependence (63%), tolerance (60%), and addiction (54%) are common (82). These concerns may contribute to care avoidance, with up to 77% of young adults with SCD reporting reluctance to engage with the healthcare system and instead managing pain at home, often suboptimally (83).

Addressing these disparities requires a multifaceted approach that includes provider education, implementation of evidence-based pain protocols, expansion of multidisciplinary care models, and policies that ensure equitable access to appropriate pain management. Efforts to reduce stigma and center patient-reported experiences are essential to improving trust, engagement, and outcomes in this vulnerable population.

### 2.13 Special considerations in the aging SCD adult

Renal dysfunction is a critical concern in older adults with SCD and necessitates careful adjustment of analgesic dosing, along with avoidance of nephrotoxic medications. Routine screening for microalbuminuria is recommended, and angiotensin-converting enzyme (ACE) inhibitors may be indicated to slow the progression of kidney disease (10, 11). Table 1 shows analgesic suitability for the older adult with sickle cell disease.

**Table 1:** Therapeutic decision-making for younger vs. older adult.

Clinical consideration	Adult in their thirties with preserved function	Adult over sixty or a frail adult
Physiological reserve	Often greater, although SCD organ damage may still be present	More likely to have reduced renal, hepatic, cardiopulmonary, and neurologic reserve
Medication clearance	May be relatively preserved	Greater risk of delayed clearance and metabolite accumulation
Pain generators	Vaso-occlusive, nociceptive, neuropathic, or nociplastic	More likely to have multiple overlapping SCD and age-associated pain generators
Opioid risk	Sedation and respiratory risks remain relevant	Greater concern for delirium, falls, fractures, constipation, and respiratory suppression
NSAID use	May be feasible for brief use if renal function is preserved	Often limited by nephropathy, bleeding, cardiovascular disease, or interacting medications
Adjuvant analgesics	Titrated according to mechanism and tolerability	Greater sensitivity to dizziness, anticholinergic effects, cognitive slowing, and gait impairment
Polypharmacy	May be less extensive	More likely to involve interacting medications and multiple prescribers
Monitoring priorities	Pain, function, adherence, and adverse effects	Also cognition, gait, falls, bowel function, orthostasis, renal function, and medication management
Treatment goals	Symptom control and participation in work/family roles	Symptom control plus independence, mobility, cognition, fall prevention, and treatment burden
Decision framework	Chronological age has limited importance	Frailty and physiological reserve may be more informative than age alone

Neurocognitive impairment is also prevalent in this population, with approximately one-quarter of older adults experiencing a history of overt stroke and up to half demonstrating silent cerebral infarcts or cortical atrophy (41). These changes may influence pain perception, reporting accuracy, and adherence to treatment, underscoring the need for cognitive assessment and supportive interventions (10).

Cardiopulmonary complications account for a substantial proportion of mortality in adults with SCD, contributing to nearly half of all deaths (14). In this context, the use of opioids requires careful monitoring due to the increased risk of respiratory depression (11).

Finally, for patients receiving long-term opioid therapy, any dose reduction should be approached cautiously. Gradual tapering is essential to minimize the risk of withdrawal, unintentional overdose, and adverse mental health outcomes (11).

## 3 Discussion

The central conclusion of this review is not that older adults with SCD develop an entirely distinct pain mechanism. Rather, aging changes the context in which established pain mechanisms arise and persist. Lifelong vaso-occlusion and hemolysis create cumulative tissue and neural injury; age-associated musculoskeletal and metabolic disorders add new pain generators; and multimorbidity, sleep disturbance, cognitive change, and reduced physiological reserve increase the functional consequences of pain (Table 2). Chronic pain in the older adult with SCD may therefore represent a progressively complex mixture of repeated episodic vaso-occlusive pain, persistent nociceptive input, neuropathic injury, and altered nociceptive processing (4, 6–9, 17, 18).

**Table 2:** Summary of analgesic suitability.

Analgesic	Suitability in Elderly SCD	Key Considerations
Acetaminophen	First-line non-opioid	Limit ≤2 g/day with hepatic disease; safe in CKD
Topical NSAIDs/	Preferred over	Minimal systemic absorption;
lidocaine	Systemic NSAIDs	safe in CKD
Duloxetine	First-line for neuropathic pain	Avoid if CrCl <30; preferred over TCAs
Gabapentin	Effective for neuropathic pain	Dose-reduce per CrCl; avoid combining with opioids
Buprenorphine	Preferred opioid in	Hepatic metabolism; ceiling
(transdermal)	CKD	on respiratory depression
Hydromorphone	Acceptable with dose adjustment	Preferred over morphine in CKD
Fentanyl	Acceptable in non-	Hepatic metabolism; avoid in
(transdermal)	dialysis CKD	dialysis
Morphine	Avoid in significant CKD	Neurotoxic metabolite accumulation
Systemic NSAIDs	Use with extreme caution	High risk of AKI, GI bleeding, cardiovascular events
TCAs (amitriptyline)	Avoid in elderly	Anticholinergic effects, falls, sedation
Meperidine	Contraindicated	Neurotoxic metabolite; avoid in all SCD patients

This narrative review highlights the growing and underrecognized burden of chronic pain among aging adults with sickle cell disease (SCD), a population that reflects the success of advances in disease-modifying therapies while simultaneously exposing critical gaps in long-term, equitable pain management. As individuals with SCD live longer, chronic pain has emerged as a dominant and persistent complication, characterized by complex, overlapping nociceptive, neuropathic, and centralized mechanisms. Importantly, this evolution from episodic to chronic pain is not only biologically driven but also shaped by cumulative organ damage, treatment exposures, and broader psychosocial and structural determinants.

This mechanistic framework has direct translational relevance. A patient whose pain is primarily driven by active tissue injury may benefit from treatment of the underlying SCD complication, disease-modifying therapy, rehabilitation, and appropriately selected analgesics. Neuropathic features may prompt a focused neurologic assessment and consideration of mechanism-targeted adjuvant medications. A prominent nociplastic or central-sensitization phenotype may support greater emphasis on graded physical activity, sleep interventions, cognitive-behavioral approaches, and other strategies intended to improve pain modulation and function. These treatments are not necessarily exclusive, because mixed pain phenotypes frequently require a coordinated multimodal plan. For aging adults, the selection of therapies must also account for renal, hepatic, cardiovascular, cognitive, and functional vulnerabilities. This mechanism-informed approach may help clinicians move beyond treating all chronic SCD pain as recurrent vaso-occlusion while avoiding the equally problematic assumption that pain without a readily identifiable structural lesion is not biologically based.

A key finding across this synthesis is that chronic pain in aging SCD is best understood as a multidimensional condition requiring integrated, patient-centered care. Pharmacologic management remains foundational; however, it is increasingly clear that reliance on analgesics alone, particularly opioids, fails to adequately address the full burden of pain. While opioids continue to play a necessary role for many patients, especially those with severe or refractory pain, their use must be balanced against risks associated with aging, including organ dysfunction, polypharmacy, and altered pharmacodynamics. Similarly, commonly used agents such as nonsteroidal anti-inflammatory drugs (NSAIDs) carry heightened risks in this population due to the high prevalence of renal and cardiovascular disease. These considerations underscore the need for individualized treatment strategies that account for both disease-specific and age-related vulnerabilities.'

Pharmacologic management is one of the areas in which an aging-focused framework most clearly changes clinical decision-making. A medication regimen that is appropriate for a younger adult with preserved renal function and limited comorbidity may carry substantially different risks in an older adult with nephropathy, cerebrovascular injury, impaired mobility, sleep-disordered breathing, or polypharmacy. Accordingly, treatment decisions should incorporate physiological reserve, frailty, cognition, fall risk, organ function, drug interactions, and patient goals rather than relying on chronological age alone.

This geriatric framework must be balanced against the longstanding undertreatment and stigmatization of SCD pain. Concerns about falls, cognitive effects, or opioid-related harm should lead to individualized selection, monitoring, and multimodal care, not reflexive withholding of analgesia or abrupt dose reduction. Shared decision-making is particularly important when the benefits of pain relief and preserved function must be weighed against sedation, delirium, falls, respiratory risk, and treatment burden.

Nonpharmacological and behavioral interventions represent essential, though underutilized, components of comprehensive pain management. Evidence supporting modalities such as cognitive behavioral therapy (CBT), physical rehabilitation, and integrative therapies remains modest but promising, particularly in addressing the emotional and functional dimensions of pain. Expanding access to these interventions is critical, especially given their low-risk profile and potential to reduce reliance on pharmacologic therapies. Future research should prioritize rigorous evaluation of these approaches in older SCD populations, where evidence is currently sparse.

An important clinical implication is that non-pharmacologic strategies should be integrated prospectively into multimodal pain care rather than reserved for patients who have “failed” pharmacologic treatment. This is particularly relevant for aging adults with SCD, in whom cumulative renal, hepatic, cardiopulmonary, neurologic, and musculoskeletal complications may increase medication-related risk. Physical rehabilitation, behavioral pain management, sleep treatment, and selected integrative approaches address functional and psychosocial dimensions of pain that analgesics alone may not adequately improve. However, offering these interventions must not become a rationale for undertreating pain, abruptly reducing necessary medication, or delaying investigation of new SCD complications (11, 32, 33).

The practical value of these strategies depends on accessibility, cultural acceptability, affordability, and continuity. Adults with SCD may encounter limited insurance coverage, transportation barriers, shortages of clinicians familiar with SCD, and stigma when behavioral treatment is incorrectly interpreted as suggesting that their pain is not biologically based. Shared decision-making is therefore essential. Telehealth, home-based rehabilitation, and digitally supported behavioral interventions may improve access, but their effectiveness and acceptability among older adults with SCD require further study.

Beyond clinical management, this review underscores the profound influence of psychosocial burden and health disparities on pain experiences and outcomes. Aging adults with SCD face disproportionately high rates of depression, anxiety, and social isolation, all of which are closely linked to increased pain severity, poorer quality of life, and greater healthcare utilization. At the same time, systemic inequities, including racial bias, stigma, and inadequate access to specialized care continue to compromise pain management. Delays in receiving analgesia, mistrust of patient-reported pain, and restrictive opioid prescribing practices illustrate how structural barriers directly translate into suboptimal care. These findings align closely with the broader aims of the Research Topic, emphasizing that pain disparities are not solely clinical issues but reflect deeper inequities within healthcare systems.

Importantly, policy-level factors have also contributed to unintended consequences in pain management for SCD. The misapplication of opioid prescribing guidelines, despite explicit exclusions for SCD, has led to reduced access to appropriate analgesia for a population with clear clinical need. This highlights the importance of disease-specific guidance and reinforces the necessity of clinician education to ensure that policies are applied appropriately and equitably.

The concept of SCD as a form of accelerated aging provides a useful framework for understanding the intersection of chronic disease and geriatric syndromes. Older adults with SCD experience earlier onset of comorbidities such as renal disease, cardiopulmonary complications, and neurocognitive impairment, all of which complicate pain assessment and management. Integrating geriatric principles—such as functional assessment, cognitive evaluation, and medication optimization—into SCD care is therefore essential to improving outcomes.

The psychosocial consequences of chronic SCD pain should be understood as dynamic across the adult life course. Depression, anxiety, stigma, and discrimination remain important, but later-life pain occurs within a changing context of retirement, bereavement, caregiver loss, functional decline, cognitive vulnerability, multimorbidity, and increasing treatment burden. These factors may reduce social participation and independence while increasing reliance on family members or formal services. Conversely, older adults may bring decades of disease knowledge, resilience, and self-advocacy that should inform shared decision-making.

Direct evidence concerning these aging-specific psychosocial trajectories in SCD remains sparse. Therefore, some proposed relationships are inferred from broader chronic-pain and gerontological literature. This gap limits the ability to determine which psychosocial changes result from chronological aging, cumulative SCD burden, disability, survivorship, or broader social conditions.

### 3.1 Limitations

Several gaps in the literature warrant attention. There is a lack of age-specific clinical trials evaluating both pharmacologic and nonpharmacologic interventions in older SCD population. The available literature does not yet permit chronological aging to be separated reliably from disease duration, cumulative pain exposure, organ damage, treatment history, survivorship, and birth-cohort effects. Older adults represented in current studies are also survivors of earlier treatment eras and may differ systematically from younger adults in disease severity, access to disease-modifying treatment, and healthcare exposure. Statements about age-specific mechanisms must therefore remain cautious. Additionally, limited data exist on long-term outcomes associated with chronic opioid therapy, multimodal pain strategies, and emerging treatments in this group. Research is also needed to better understand the biological mechanisms underlying chronic pain in the aging patient with SCD, including the roles of inflammation, neuroplasticity, and psychosocial stressors.

Several limitations inherent to the narrative-review design should be considered. Although explicit search, eligibility, and evidence-prioritization procedures were used to improve transparency, study identification, selection, and interpretation involved author judgment and may therefore be susceptible to selection bias, incomplete retrieval of relevant evidence, and subjective weighting of findings.

## 4 Conclusion

In conclusion, chronic pain in aging adults with sickle cell disease (SCD) represents a complex and evolving clinical challenge that reflects both advances in survival and persistent gaps in equitable care. It is best understood as the product of lifelong disease exposure interacting with biological aging, cumulative organ damage, age-associated comorbidity, and declining physiological reserve. Aging does not necessarily create a unique pain mechanism; rather, it changes the number, relative importance, persistence, and consequences of nociceptive, neuropathic, and nociplastic processes. An age-informed approach must therefore move beyond treating all pain as recurrent vaso-occlusion and incorporate structural injury, neurologic disease, multimorbidity, physical and cognitive function, medication vulnerability, and patient-centered goals. As this population grows, the burden of chronic, multifactorial pain driven by cumulative organ damage, neurobiological changes, and psychosocial factors demands a shift from episodic, acute-focused management to comprehensive, longitudinal care models.

Effective management requires a multimodal and individualized approach that integrates pharmacologic therapies with nonpharmacological and behavioral interventions, while carefully accounting for age-related comorbidities, organ dysfunction, and polypharmacy. Equally important is the recognition that pain in SCD is deeply influenced by psychosocial burden and structural inequities, including stigma, bias, and barriers to timely and appropriate treatment. These factors not only exacerbate suffering but also undermine trust in the healthcare system and contribute to care avoidance.

Addressing chronic pain in this population necessitates alignment with broader efforts to advance equity in geriatric pain management. This includes improving provider education, expanding access to multidisciplinary care, ensuring appropriate application of clinical guidelines, and prioritizing patient-centered outcomes.

In addition to improving on current knowledge of opioid management in this aging population, future studies should prioritize larger, well-designed clinical trials evaluating alternatives to opioid therapy, particularly those incorporating outcome measures that capture nociplastic pain and central sensitization, which are increasingly recognized features of chronic SCD pain (65). In parallel, there is a need to develop and standardize manualized cognitive behavioral therapy (CBT) protocols tailored specifically to individuals with SCD across the lifespan, to improve scalability and implementation in both clinical and community settings (11).

Ultimately, improving outcomes for aging adults with SCD will require a coordinated, equity-focused approach that bridges clinical innovation with systemic change, ensuring that all patients receive compassionate, evidence-informed, and effective pain management across the lifespan.
